# Supplementary material for: Analysis of PPARγ Signaling Activity in Psoriasis
Source: Int J Mol Sci. 2021 Aug 10;22(16):8603. doi: 10.3390/ijms22168603 (PMC8395241; doi:10.3390/ijms22168603)
Supplement: Supplementary file 1 [file ijms-22-08603-s001.zip › Supplemental materials_Analysis of PPARg signaling activity in psoriasis/Pathway models/Models images and html files/Interleukin-17 and interleukin-22 signaling in psoriasis/6835.html]

KRT17


# Protein KRT17

|  |  |
| --- | --- |
| URN | urn:agi-llid:3872 |
| Total Entities | 0 |
| Connectivity | 613 |
| Name | KRT17 |
| Description | keratin 17 |
| Notes | This gene encodes the type I intermediate filament chain keratin 17, expressed in nail bed, hair follicle, sebaceous glands, and other epidermal appendages. Mutations in this gene lead to Jackson-Lawler type pachyonychia congenita and steatocystoma multiplex. [provided by RefSeq, Aug 2008] |
| Primary Cell Localization | Cytoplasm |

---

|  |  |
| --- | --- |
| Pathway | Proteins Involved in Psoriasis |
|  | IL22 Induces Keratinocyte Proliferation in Psoriasis |
|  | Proteins with Altered Expression in Psoriasis |
|  | Dioxin Induced Chloracne (Hypothesis) |
|  | 2\_Interleukin-17 and interleukin-22 signaling in psoriasis |
|  | Interleukin-17 and interleukin-22 signaling in psoriasis |

---

|  |  |
| --- | --- |
| MedScan ID | 3872 |

---

|  |  |
| --- | --- |
| LocusLink ID | 3872 |
|  | 16667 |
|  | 287702 |

---

|  |  |
| --- | --- |
| Alias | Krt1-17 |
|  | pachyonychia congenita 1 (Jackson-Lawler type) |
|  | CK 17 |
|  | type I keratin KA17 |
|  | pachyonychia congenita I gene |
|  | pachyonychia congenita I protein |
|  | 2410039E07 |
|  | keratin, type I cytoskeletal 17 |
|  | K17 |
|  | keratin 17 epitope S2 |
|  | pachyonychia congenita 1 protein |
|  | RP23-217I3.9 |
|  | keratin complex 1, acidic, gene 17 |
|  | keratin 17 |
|  | keratin 17 epitope S4 |
|  | cytokeratin K17 |
|  | OTTMUSP00000002397 |
|  | PCHC1 |
|  | KA17 |
|  | PCHC I |
|  | keratin 17 epitope S1 |
|  | KRT17 |
|  | keratin 17, type I |
|  | keratin K17 |
|  | cytokeratin 17 |
|  | pachyonychia congenita 1 gene |
|  | PC |
|  | PC2 |
|  | 39.1 |
|  | CK-17 |
|  | cytokeratin-17 |
|  | keratin-17 |

---

|  |  |
| --- | --- |
| GO ID | 0042289 |
|  | 0032395 |
|  | 0005200 |
|  | 0070268 |
|  | 0008544 |
|  | 0031069 |
|  | 0045109 |
|  | 0031424 |
|  | 0030307 |
|  | 0051798 |
|  | 0045727 |
|  | 0007165 |
|  | 0071944 |
|  | 0005829 |
|  | 0070062 |
|  | 0005882 |
|  | 0045111 |
|  | 0005198 |
|  | 0002009 |
|  | 0005737 |
|  | 0045103 |
|  | 0045095 |

---

|  |  |
| --- | --- |
| KEGG ID | hsa:3872 |
|  | mmu:16667 |
|  | rno:287702 |

---

|  |  |
| --- | --- |
| Organism | Homo sapiens {Organism urn:agi-taxid:9606} |
|  | Mus musculus {Organism urn:agi-taxid:10090} |
|  | Rattus norvegicus {Organism urn:agi-taxid:10116} |
|  | Homo sapiens |
|  | Mus musculus |
|  | Rattus norvegicus |

---

|  |  |
| --- | --- |
| Mouse chromosome position | 11 63.44 cM |

---

|  |  |
| --- | --- |
| OMIM ID | 148069 |
|  | 167210 |
|  | 184500 |

---

|  |  |
| --- | --- |
| Rat chromosome position | 10q31 |
|  | 10q32.1 |

---

|  |  |
| --- | --- |
| Hugo ID | 6427 |

---

|  |  |
| --- | --- |
| Human chromosome position | 17q21.2 |

---

|  |  |
| --- | --- |
| Swiss-Prot Accession | Q04695 |
|  | Q14666 |
|  | Q04695.2 |
|  | Q9QWL7 |
|  | Q9QWL7.3 |
|  | Q6IFU8 |
|  | Q6IFU8.1 |
|  | A5Z1M9 |
|  | A5Z1N0 |
|  | A5Z1N1 |
|  | A5Z1N2 |
|  | A6NDV6 |
|  | A6NKQ2 |
|  | Q6IP98 |
|  | Q8N1P6 |
|  | A2A4G6 |
|  | Q61783 |

---

|  |  |
| --- | --- |
| PIR ID | S30433 |
|  | C26135 |

---

|  |  |
| --- | --- |
| GenBank ID | NC\_000017 |
|  | NM\_000422 |
|  | NP\_000413 |
|  | NG\_008625 |
|  | NG\_009090 |
|  | AC130686 |
|  | CH471152 |
|  | EAW60750 |
|  | EAW60751 |
|  | EAW60752 |
|  | EAW60753 |
|  | S78515 |
|  | AAB34565 |
|  | S81026 |
|  | AAD15003 |
|  | Z19574 |
|  | CAA79626 |
|  | Z31596 |
|  | AK095342 |
|  | BAC04534 |
|  | AK304373 |
|  | BAG65211 |
|  | BC000159 |
|  | AAH00159 |
|  | BC011901 |
|  | AAH11901 |
|  | BC056421 |
|  | AAH56421 |
|  | BC072018 |
|  | AAH72018 |
|  | BC072019 |
|  | AAH72019 |
|  | BP226061 |
|  | BX647923 |
|  | EF608068 |
|  | ABQ96595 |
|  | EF608069 |
|  | ABQ96596 |
|  | EF608070 |
|  | ABQ96597 |
|  | EF608071 |
|  | ABQ96598 |
|  | X05803 |
|  | CAA29248 |
|  | X62571 |
|  | CAA44451 |
|  | Q04695 |
|  | NC\_000077 |
|  | NM\_010663 |
|  | NP\_034793 |
|  | AL590873 |
|  | CH466662 |
|  | EDL02571 |
|  | AB013608 |
|  | BAA34229 |
|  | BC032161 |
|  | BC132454 |
|  | AAI32455 |
|  | BC132456 |
|  | AAI32457 |
|  | EH104192 |
|  | EH105154 |
|  | M13805 |
|  | AAA39394 |
|  | Q9QWL7 |
|  | NC\_005109 |
|  | NM\_212545 |
|  | NP\_997710 |
|  | AC\_000078 |
|  | AAHX01065996 |
|  | AC096895 |
|  | CH473948 |
|  | EDM06025 |
|  | BC100058 |
|  | AAI00059 |
|  | BK004050 |
|  | DAA04484 |
|  | Q6IFU8 |
|  | NC\_018928 |
|  | AMYH02033411 |
|  | DQ891101 |
|  | ABM82027 |
|  | DQ894283 |
|  | ABM85209 |
|  | AC\_000033 |
|  | AAHY01095746 |

---

|  |  |
| --- | --- |
| Swiss-Prot ID | K1C17\_HUMAN |
|  | K1C17\_MOUSE |
|  | K1C17\_RAT |

---

|  |  |
| --- | --- |
| Cell Localization | Cytoplasm |

---

|  |  |
| --- | --- |
| Ensembl ID | ENSG00000128422 |
|  | ENSP00000308452.8 |
|  | ENST00000311208.13 |
|  | ENSMUSG00000035557 |
|  | ENSMUSP00000079699.6 |
|  | ENSMUST00000080893.6 |
|  | ENSRNOG00000026371 |
|  | ENSRNOP00000005382.5 |
|  | ENSRNOT00000005382.6 |
|  | ENSP00000308452 |
|  | ENST00000311208 |
|  | ENSMUSP00000079699 |
|  | ENSMUST00000080893 |
|  | ENSRNOP00000005382 |
|  | ENSRNOT00000005382 |

---

|  |  |
| --- | --- |
| MGI ID | MGI:96691 |

---

|  |  |
| --- | --- |
| RGD ID | 1303181 |

---

|  |  |
| --- | --- |
| Unigene ID | Hs.2785 |
|  | Mm.14046 |
|  | Rn.106755 |

---

|  |  |
| --- | --- |
| Homologene ID | 363 |

---
